# Study Protocol: 6th National Survey of Australian Secondary Students and Adolescent Sexual Health, 2018

**DOI:** 10.3389/fpubh.2019.00217

**Published:** 2019-08-22

**Authors:** Christopher Fisher, Gosia Mikolajczak, Paulina Ezer, Lucille Kerr, Rosalind Bellamy, Graham Brown, Andrea Waling, Jayne Lucke

**Affiliations:** ^1^Australian Research Centre in Sex, Health and Society, La Trobe University, Melbourne, VIC, Australia; ^2^The Policy Lab, School of Social and Political Sciences, The University of Melbourne, Melbourne, VIC, Australia

**Keywords:** adolescents, HIV, sexually transmitted infections, sex education, survey

## Abstract

**Introduction:** Since 1992 the Australian Government has funded a periodic national survey of HIV and Sexually Transmissible Infection (STI) knowledge and sexual risk behavior among secondary school students. Adolescents continue to be a priority population in public health efforts to reduce rates of STIs in Australia. The purpose of the survey is to inform progress on national strategic sexual health priorities. The results are used by federal and state/territory government agencies, youth-serving community organizations and health educators to improve knowledge, promote healthy sexual behaviors and target educational efforts aimed at communicating public health messages to young people.

**Materials and Equipment:** The 6th survey entitled the “National Survey of Secondary Students and Adolescent Sexual Health” was conducted online in 2018 among 14–18 year olds living in Australia. The anonymous self-complete survey contained up to 286 items assessing three primary domains of knowledge, behaviors and education experiences. Factual knowledge measures covered HIV transmission and STI knowledge around transmission and prevention covering gonorrhea, Chlamydia, syphilis, hepatitis, herpes, and HPV. Behavioral measures examined perceived susceptibility, peer norms, protective behaviors, age of onset for various behaviors, reasons for not being sexually active yet, and/or sexual histories with additional detail on most recent sexual event. The 6th survey was completed by 8,400 Australian adolescents a represents a broad cross-section by age, gender, year in school, type of school (e.g., government, Catholic), and state/territory which closely matched census data on these strata. The one-of-a-kind survey instrument, grounded in public health theories, may prove valuable for public health researchers.

**Expected Impact of the Study on Public Health:** Findings from the 6th National Survey of Secondary Students and Adolescent Sexual Health will contribute important insights into current knowledge, behaviors and educational experiences of young people. Results, similar to previous iterations of the survey, will inform public health practitioners, policymakers, educators, and advocates for the sexual health and well-being of young Australians. Results may assist sexual health services to align with broader public health goals articulated in the national HIV and STI strategies aimed to reduce the burden of disease and improve the quality of sexual lives of young Australians.

## Introduction

### Background and Rationale

In Australia, adolescents continue to be a priority population for sexual health promotion (1, 2). Nationally, overall population level infection rates of the three major Sexually Transmissible Infections (STIs), Chlamydia, gonorrhea and syphilis, rose significantly over the past 5 years at 13, 80, and 135%, respectively (3) with adolescents accounting for a disproportionate burden of disease. Australian adolescents and young adults aged 15–29 years accounted for three-quarters (73%) of Chlamydia notifications in 2017 (3). Young women bear a disproportionate burden of gonorrhea with 15 to 19-year-old females experiencing nearly three times the rate of infection of all women (3). The recently released 2018–2022 Australia National STI Strategy seeks to address adolescent sexual health issues, in part, through continued and improved monitoring of key determinants associated with adolescent sexual health, including knowledge, behavior, and sexual health education (1).

Knowledge serves as a key distal determinant of behavior in a number of health behavior theories and models, including the Health Belief Model (4), Theories of Reasoned Action and Planned Behavior (5, 6), Social Cognitive Theory (7), and the Stages of Change Model (8). Modifying behavior to mitigate negative sexual health outcomes such as STIs requires accurate knowledge of prevention, transmission, testing, and treatment. For example, accurate knowledge of condom efficacy has been shown to influence the perceived benefits of using a condom for prevention in the Health Belief Model (9, 10). Ongoing surveillance of knowledge levels among adolescents serves to identify points of focus, such as knowledge of symptoms of an STI, for future public health information campaigns, interventions, and program curricula promoting healthy sexual behaviors.

Behavior modification to improve sexual health outcomes, regardless of the theory or theories used, depends on knowing which behaviors young people are currently engaging in, as well as the context in which they occur (11). Data on sexual practices of young people coupled with context allows for targeted and nuanced messages to bolster changes in proximal determinants of health behavior theories. For example, sociodemographic data from previous studies have shown older adolescents may be less likely to be using condoms (12), indicating a need for programs and interventions designed to increase condom use and/or ensure that other protective factors (e.g., hormonal contraception) take their place. Recent technological changes are reshaping human interactions, including those related to sexual behavior (e.g., sexting) and well-being (e.g., cyberbullying). Due to their prevalence and the potential consequences for sexual and mental health, these newer behaviors require to be tracked. Surveillance data on technology-related behaviors can inform health education interventions and curriculum by not only illuminating prevalence of behaviors but also documenting the impacts of engaging in such behaviors at all levels of the Social Ecological Model (13).

Education serves as the primary tool of instilling young people with knowledge to influence their sexual behaviors or practices in the ongoing campaign to reduce negative sexual health outcomes. Formal channels, such as school-based sexuality and relationships education, have been shown, dependent on many factors such as dosage and delivery style, to have some impact on behaviors, practices and ultimately outcomes (14). Less formal channels, including “The Talk” with parents, seeking information on the Internet, and talking with peers, show similar variations in efficacy to improve sexual health. Tracking the formal and informal sources young people currently use, find useful and trust are vital for identifying where and through whom to communicate sexual health and well-being messaging.

In Australia, the challenge of addressing adolescent sexual health is further complicated by a fast growing and increasingly diverse population, driven primarily through migration (15). Between the 2011 and 2016 census the population grew by 1.9 million, an 8.8% increase. Immigration drove the bulk of this increase with the majority arriving from New Zealand, China and India (16).The increasing social and cultural diversity of Australia justifies a need for ongoing surveillance of likely shifting knowledge, behavior and education in relation to adolescent sexual health and well-being.

The dynamic nature of the Australian population, technological advances, and the resulting cultural shifts impact on young people's sexual health knowledge, behaviors and practices, and the education that informs them. Research, as noted in the recent National Strategy, continues to be a vital part of informing public health and education efforts to ameliorate the increasing negative sexual health outcomes facing Australian youth and young adults.

### The Study

The Australian Government has funded a periodic national survey of HIV and STI knowledge, sexual behavior, and informal sexual health education (e.g., sources of information) of secondary school students since 1992, adding formal sexual education measures in 2013. The purpose of the periodic surveys have been to inform progress on national strategic sexual health priorities, particularly relating to the level of knowledge about the transmission of HIV, STIs and rates of sexual behaviors.

The survey was initiated amid concerns about the vulnerability of young people to HIV infection and the sense that both health and education authorities needed a more realistic picture of the knowledge and behaviors of young people if effective prevention was to be undertaken (17, 18). The data collected throughout the five iterations of the survey have given a robust picture of the sexual health knowledge, attitudes, beliefs, and practices of Australian young people. The findings of these surveys have been widely used throughout Australia and have been relied upon, over the last 25 years, to guide the work of health professionals, teachers, youth workers, service planners, and policymakers. Survey results have been used to inform educational policy and sexual health programs, to improve the relevance of sexual health resources available to teachers, and by health departments to plan interventions for young people in Australia. Results have been published in reports (19) that have been made available to schools and education authorities, as well as in academic journals. The reports have been used as the basis for the development of classroom resources for sexuality education and health promotion materials for young people (e.g., lovesexrelationships.edu.au). Survey findings have also been used to provide an indicator of the success of the National HIV/STI Strategies, and as the basis of many other state and territory policies and plans for supporting the sexual health of young people.

Previous research impacts from the first five surveys, as noted above, were made possible through inclusion of the following key topics:

- Students' knowledge of HIV/AIDS, STIs, HPV, hepatitis;
- Students' sexual behavior and experiences;
- Students' sexual attitudes and feelings;
- Students' use of the Internet, technology, and social media related to sexual health and relationships; and,
- Experiences of sexual health and relationships education, both formal and informal.

The 6th survey, entitled the “National Survey for Secondary Students and Adolescent Sexual Health,” was conducted in 2018 among 14–18 year olds living in Australia. Similar to previous iterations, it examined knowledge about HIV and other STIs, sexual behavior (including sexting), and experiences of sexuality and relationships education, both formal and informal. While the 2013 survey collected additional data via an online survey to boost numbers following difficulties recruiting using only the traditional school-based paper and pen survey methodology (19), the 2018 survey used an exclusively online survey format for the first time. In addition, the 2018 survey was crafted to be completed by participants in a short amount of time (~20 min), but to retain as many questions from previous surveys as possible for comparison purposes while responding to new and emerging issues. This paper presents the study protocol and participant characteristics of the 2018 survey.

### Aims

The aims of the survey are:

1. To examine the knowledge, attitudes, beliefs and practices of Australian adolescents aged 14–18 years in relation to sexual health, including knowledge of HIV, sexually transmitted diseases, and blood-borne viruses.
2. Where the same or very similar questions are asked, to compare the results of the 2018 survey with those of the 1992, 1997, 2002, 2008, and 2013 surveys to provide evidence of change in the sexual health knowledge, attitudes, beliefs, and practices of young Australians.
3. To disseminate survey findings via a published report, public presentations and other academic and public channels in order to enable government agencies and community-based organizations to develop appropriate interventions that enhance the sexual health and well-being of young people.

## Methods And Analysis

### Materials and Equipment

#### Consultation Stage

Extensive consultation with key stakeholders informed the development of the present survey instrument and protocol in line with principles of community-engaged research (20). End users (*N* = 49) of the results such as education and health departments, community-based organizations (e.g., family planning), policymakers, researchers, and teachers formed the community of interest for consultation. While parent groups were not specifically included in the end users group given results are predominately aimed at professionals in the field of adolescent sexual health, many of those consulted self-identified as parents. One-on-one conversations about the survey's content domains and recruitment options were held with leaders (e.g., head of STI section of a state department of health) from the various end users and across all states and territories of Australia. A broader list of stakeholders (e.g., community-based organizations' sexuality education team) was then invited to provide similar feedback through an online portal. The dominant themes that emerged and cut across all or most consultations indicated that: (1) the results from the past iterations of the study were widely used within policy and program planning across government, education, health, and community organizations, (2) all content domains from previous versions of the survey were considered important and should be kept if possible (consultees understood the space limitations of surveys), (3) given the increasing burden on school teachers and administrators combined with the pervasiveness of internet access among young people in Australia, an online-only survey was the preferred and more feasible option, and (4) all partners indicated strong support and need for the survey and were willing to work alongside the research team to promote it. Paraphrasing one participant, “I completely understand the need to research all those topics. But, you'll never get that survey into my school. However, we could work with you to let students know about it outside of the school.”

The survey, as well as its past iterations, was funded by the Commonwealth of Australia Department of Health. While the funders were consulted and kept informed about the progress and outcomes of the research, they did not have any influence over the methods or findings.

#### Survey Instrument/Measures

All items had a forced-choice format (i.e., respondents had to answer an item to continue with the survey). A “Prefer Not to Answer” option was provided to ensure participants could “opt out” of any one question they felt uncomfortable answering. Given the ongoing periodic collection of data for the survey since 1992, wherever possible, the same question wordings were used to facilitate continued opportunities for multi-wave comparisons. The full survey instrument including response options can be found in the Appendix (Supplementary Material) of this article. Broadly, survey items covered four domains: socio-demographic information, knowledge, behaviors and education.

### Sociodemographics

Sociodemographics were measured using standard census-type items (21) including age, gender, year in school, school type (government, catholic, other non-government), school make-up (all boys, all girls, mixed, home schooled), place of residence (approximated with a post-code), Indigenous status, and religion. Due to ongoing high levels of immigration and the subsequent cultural and linguistic diversity in Australia, the survey also asked about country of birth (including length of residency if not born in Australia), parental country of birth (don't know options were included), and language(s) spoken at home. Additional demographics on sexual orientation and gender identity were ascertained with five standardized items developed internationally by the UCLA Williams Institute (22).

## Knowledge

### HIV Knowledge

Previous research has documented the associations between prevention behaviors and factual knowledge about HIV transmission, prevention and the associated stigma/myths (23, 24). Since 1992, the Secondary Student Survey has sought to document HIV knowledge through a set of 11 items, similar to validated survey items used in other studies (25); the 2018 survey used the same true/false questions.

### STI Knowledge

Similar to HIV, associations between knowledge and preventative behaviors has been documented in other research (26). Within the Australian context, a prolonged national focus on reductions in hepatitis infections (27, 28) and a focus on high HPV vaccination rates (29) supported the need to measure these STI knowledge sub-domains. The domain of STI knowledge was comprised of 40 items drawn from previous versions of the survey covering transmission/prevention, symptoms, impacts of STIs, and treatment. Sixteen questions focused on traditional STIs (e.g., gonorrhea), nine items on hepatitis A, B, & C, and 15 items on HPV knowledge.

## Behavior

### Perceived Susceptibility

Perceptions of susceptibility or risk of contracting HIV and/or STIs, as documented in previous research correlates with preventative behaviors (30). Four items covered perceived likelihood of getting HIV, any STI, and hepatitis B and C.

### Protective Behaviors

Protective behaviors for preventing HIV and/or STI transmission include protection (e.g., vaccination, condom use) and minimizing transmission (e.g., testing/awareness of infection). Four items covered vaccination and three items covered testing behaviors with follow-up questions on diagnosis for those indicating a positive test result.

### Peer Norms

In line with the literature on the importance of peer norms and condom use (31), two items assessed if participants thought condoms were commonly used among people their own age, and gender norms related to initiating condom use.

### Sexual Activity

Prior to asking a series of questions on behavior, participants were asked contextual questions about dating, through two items. Research in sexual behavior/practice is fraught with difficulty in defining what “having sex” means (32, 33). Participants were asked, “Have you ever had sex?” The question was then followed with a list of eight behaviors spanning from deep kissing through to oral, anal and vaginal sex. For each behavior, participants were asked to indicate at what age they first experienced it (never, under 14, 14 up through 18). Participants indicating they had not yet experienced anal or vaginal sex were redirected to a suvey section on reasons why they had not yet had “sex,” while the remaining “sexually active” participants were sent to a series of questions about their sexual experiences. For the purpose of the study, sex was defined as intercourse behaviors (i.e., penetration of the anus or vagina) as these behaviors account for the vast majority of STI and HIV infections (34, 35) which is the primary focus of the study and the rationale for which it is funded by the Commonwealth of Australia.

### No Sexual Intercourse

Participants who indicated they had not yet had sexual intercourse (either anal or vaginal), were redirected to a series of questions on reasons they had not yet had intercourse. Items, adapted from the 2013 survey, were modified from existing scales (36–38). Seventeen items asked participants to rate the importance of various reasons for not yet engaging in intercourse (e.g., I do not feel ready, it's against my religious beliefs, fear of damaging reputation). Likelihood of engaging in intercourse (anal or vaginal) in the next year and before marriage, were also measured. Relationship status and opportunity were also assessed as potential precursors to sexual intercourse. Ten affect statements around how participants felt about not yet having had sex were assessed (“Regarding not having experienced vaginal or anal sex, to what extent do you feel…happy, proud, embarrassed, etc.?”). The question mirrored a similar affect question for how sexually experienced participants felt about their last sexual encounter. Finally, four items assessed perceived social pressures from partners, friends, and parents to have sex or remain a virgin.

### Had Sexual Intercourse

Participants indicating they had engaged in sexual intercourse (anal or vaginal), were asked a series of questions on their sexual histories. Questions were adapted from previous versions of the survey. Condom use was assessed across up to eight items, four being follow-up questions based on initial answers. Condom use over the past year was assessed on a 5-point scale (never to always) with follow-ups asked regarding condom use the first time they had anal and/or vaginal sex. In relation to the most recent sexual experience, condom use discussions with the partner, availability, and actual use were measured. If a condom was not used at the last sexual encounter, a list of possible reasons were provided to ascertain why. Finally, for those indicating vaginal sex experience, a check all that apply question asked which form(s) of contraception they used (e.g., the pill, withdrawal, injection, implant), with condom as one of the options; this question doubled as a reliability check for condom use at last event for those engaging in vaginal sex.

One question about overall sexual (anal/vaginal) experiences was about ever having an unwanted sexual experience (yes/no); for those indicating “yes,” a follow up check all that apply questions sought possible reasons for the unwanted sex (e.g., too drunk at the time, being frightened, other). Another general question for those having experienced vaginal sex asked if any such event had ever resulted in a pregnancy (yes/no/don't know) and if so, whether it had been planned (yes/no). Other general recent sexual history questions asked about the gender of recent partners (only males, only females, both) and number of partners.

The remaining sexual behavior questions for sexually active adolescents focused on the last sexual experience to minimize recall bias (39). Questions covered status (e.g., just met, known for a while, current boyfriend/girlfriend), gender and age of the sexual partner, when the event occurred (e.g., in last week, 1–3 weeks ago, over 12 months ago), where the event took place (e.g., participant's house, partner's house, in a car), the conversations had prior to engaging in the intercourse (e.g., avoiding pregnancy, getting pleasure without penetration, having sex), if the participant was drunk or high at the time, and if the last intercourse was wanted by the respondent. Ten affect statements around how participants felt about the last sexual event were assessed (“The last time you had vaginal or anal sex, to what extent did you feel…happy, proud, embarrassed, etc.?”).

### Technology Use Behaviors

Social media use over 2 months prior to the survey was assessed through a check all that apply question (e.g., Facebook, Twitter, Tumblr, Dating Apps such as Tinder). For each platform ticked, standardized follow-up questions were asked on how often respondents used them (40).

Occurrence of sexting behaviors in the past 2 months prior to the survey was measured using six standardized items [e.g., sent/received sexually explicit written text messages (41)]. For yes answers, a follow-up to each statement assessed how often the behavior occurred and whom did it involve (e.g., boy/girlfriend).

Cyberbullying experience questions, similar to sexting behaviors, began with a check all that apply [e.g., sent threatening emails, received nasty text messages, deliberately ignored or left out; (41)] in the 2 months prior to the survey. For each item selected, participants responded to follow-up questions on how often the behavior occurred (5-point Likert scale; 1 = once a day or more; 3 = about once a week, 5 = only once in past 2 month).

## Education

### Informal Education

Many interventions highlight empowerment of young people to engage in informal education through information seeking (42). Such empowerment requires a young person to have the self-efficacy, or confidence, to seek information, trust in the source of information, and a willingness to regularly engage with the source (e.g., frequency). Confidence to consult various sources of sexual health information (avoiding HIV and other STIs, contraception decision-making, and sex in general) was measured across 10 potential sources (e.g., GP, mother, sibling, peer, teacher, internet website, and other with fill in the blank). Trustworthiness of 14 sources to provide accurate sexual health information was assessed. Participants were also asked to indicate if they had ever used the same 14 sources. For sources used, participants responded to follow-up questions on how often they had been used in the past year.

### Formal Education

A number of studies highlight the importance of relationships and sexuality education in ameliorating negative sexual health outcomes (43). Participants were asked if they had ever had relationships and sexuality education at school. Those indicating “yes” they had received relationships and sexuality education were asked a follow-up question on the year or years in school in which they had received it. Additional follow-up questions asked about the last time respondents had relationships and sexuality education; in particular, in which subject it was taught (e.g., health and physical education, science/biology), who taught it (e.g., teacher, school nurse, outside person), and how relevant they found the classes in general. A final question to all participants offered a space to write, in their own words about their sexuality education at their school (e.g., how useful it had been).

### Stepwise Procedures

#### Cross-Sectional Study Design and Sample Size

The 6th National Survey of Secondary Students and Adolescent Sexual Health is a cross-sectional survey of young people living in Australia, which is part of a series of repeated cross-sectional surveys of the adolescent population that began in 1992. Data for the 6th survey were collected between April and May 2018 via an anonymous online survey instrument containing between 220 and 286 items, dependent on skip logic and re-direction patterns, as described above. The survey was voluntary and a “Prefer Not to Answer” option was made available to participants for every question.

Since 1992, the surveys have obtained sample sizes of between 1,741 and 3,550 participants (19, 44–47). A goal of 3,239 participants was planned through minimum quota sampling (48) based on two sets of strata with medium effect sizes, both based on the most recent census (ABS, 2016). The first strata encompassed year in school covering years 10 and 12 (i.e., 10th and 12th grade or grade 10 and 12). Estimates were based on the total 2016 Australian student population in years 8 and 10 as these students would be in years 10 and 12 at the time of data collection in 2018 (school years in Australia begin in February/March and end in December of each calendar year). Initial year in school strata covered year 10 and 12 as these groups are consistent across all iterations of the survey. The first strata also included gender (female/male; transgender and gender diverse were excluded due to a lack of reliable estimates/data), and school type (government, catholic and other non-government schools; each enroll a substantive number of students in Australia). The second, independent strata included state and territory census data, with an oversample of twice the minimum quota for the smaller populations of Northern Territory, Australian Capital Territory, and Tasmania. The two independent strata provided reasonable minimum quotas whereas if they had been combined into one strata, they would have required an extremely large minimum sample (*N* = 91,112) for a medium effect size.

### Recruitment

#### Inclusion/Exclusion Criteria

Participants had to be between the ages of 14 and 18 years of age in order to participate in the survey given that the aim of the study was to report on sexual health knowledge and practices of high school adolescents, particularly those in years 10 and 12. The age range allowed for capturing almost all possible year 10 and 12 students. Participants needed to live in Australia. Participants not between 14 and 18 years of age and/or not living in Australia were excluded from participating in the survey.

#### Recruitment Strategy

This research project used a mixed methods (mostly quantitative with a few qualitative items) anonymous online survey. The study cohort was recruited by using a two-phase recruitment strategy, described as follows:

**Phase 1:** Participants were informed of the study through Facebook advertising. Social media use research suggests 93% of 18–29 year olds in Australia currently have a Facebook profile and access it an average of 16 times a week. This is higher than any other age group. Further, Australians, on average, spend 10 h a week on Facebook (40). Advertisements for the survey appeared to any Facebook user whose profile identified them as “living in Australia” and between the “ages of 14–18 years,” a reach of ~1,300,000 potential participants. The Facebook ads did not provide for interactive communication with potential participants; they could initiate communication via an e-mail link on the survey website. Adolescents interested in learning more about the survey were able to click on the advertisement itself, which then opened the survey homepage. The homepage contained a link to the survey at the top of the page, the Participant Information Statement (PIS) text in an “About the Survey” page, a resources page, FAQ for parents and teachers, and links to previous study reports. Potential participants were able to click the link to start the survey or explore the website to learn more about the study. Upon clicking to begin the survey, participants were able to read a description of the study, the requirements for participation, the time commitment for completing the survey, and other relevant details about the research.
  During Phase 1 of recruitment, the Facebook advertising strategy was refined so that advertisements were directed to potential participants based on established minimum quotas. That is, advertisements were directed toward sub-cohorts where quotas had not yet been met. For example, if fewer males than females were completing the survey, the Facebook advertisement was displayed more often to male adolescents until the quota for males was reached.
**Phase 2:** The second phase of recruitment was planned for implementation ~1 month after data collection begun, in the case that participant quotas had not been met by this time. This back-up plan was necessary because of previous difficulties in recruiting adequate numbers of participants (19). In fact, participant quotas were met through Phase 1 recruitment without needing to move to Phase 2. If required it had been planned to send out an email requesting assistance with participant recruitment to a wide range of contacts, such as Family Planning organizations, education groups, teachers, and other contacts within the education and sexual health sectors. Nationwide consultation, as noted above, indicated that these contacts would be amenable in advertising the survey through their own networks, for example on organization-specific Facebook pages, online forums, and/or newsletters, generating interest in the survey through these avenues.

### Procedure

Potential participants who visited the survey website were greeted by the survey homepage, which contained links to start the survey. The first page of the survey was the Participant Information Sheet which described the study in detail and specified eligibility criteria. The Human Ethics Committee approved the information provided including supporting that it was written at a level appropriate for the age of the potential participants; additionally, no queries to explain the information sheet were received. Potential participants were informed from the outset that participation in the survey was voluntary and that they could choose not to answer any given question by selecting the “Prefer Not to Answer” option and/or exit the survey by simply closing or redirecting their browser at any time.

Subsequent to reading the information, participants could choose to proceed to the survey itself by clicking “I AGREE,” the act of which was taken as confirmation of their assent/consent to participate in the study. The survey then asked a series of screening questions to determine eligibility. Participants were unable to proceed to the next part of the survey unless they met the inclusion criteria of living in Australia and being aged between 14 and 18 years. If they met these inclusion criteria, they were asked to complete the survey, which was estimated to take 20 min. Answers to questions were captured as the participant moved through each page of the survey.

Participants were able to resume the survey from the same device and browser for a period of 24 h in case of internet connection failure or if they needed to pause the survey. After 24 h, participants were required to re-start the survey. A footer at the bottom of each page of the survey reminded the participant that they could choose to exit the survey at any time, and included a hyperlink to the Kids Helpline and Lifeline webpages and toll-free phone numbers for anyone who may have been feeling distressed.

Upon completion of the survey, participants were directed to a final thank you and prize draw entry page which was separate from the survey in order to protect respondents' anonymity. This page again listed contact details for Kids Helpline and Lifeline (phone numbers and URLs) as well as hyperlinks to the survey website resources page which contained links to local state/territory Family Planning organization websites and sexual health organizations which maintain up-to-date sexual health information, advice and services. These additional measures were taken in order to mitigate any potential distress and minimize risk/harm to participants.

On the final thank you page, participants were asked if they wished to enter into the draw for 1 of 20 $100 Visa gift cards by entering their email address or typing “NO.” They were then asked to confirm their email address or to re-type “NO” if not entering the draw. If an email address was provided, participants were asked if they would be willing to be contacted again via the email provided for future research. Participants were informed on the Participant Information Sheet and again on the draw entry page that all emails were kept in a separate password protected file with no identifiers that could be connected to the participants' survey responses.

### Data Analysis

Detailed cross-sectional findings will be reported for the 2018 survey, and, where possible, compared to those of 1992, 1997, 2002, 2008, and 2013 to identify trends and meaningful differences. Statistical analyses will be used as appropriate and may include non-parametric options, such as chisquare and parametric alternatives, such as correlation, ANOVA, logistic regression, and other multivariate analyses. Qualitative responses will be analyzed using standard thematic analysis procedures.

### Ethics

The protocol was approved by the La Trobe University Human Ethics Committee (HEC18030). La Trobe University subscribes to and strictly adheres to the highest ethical standards in conducting research as laid out by the Australian government agency, the National Health and Medical Research Council (49).

### Consent

Due to the online format of the survey, it was not possible to obtain consent from parents/guardians. Although the Participant Information Sheet recommended that people under 18 should discuss taking the survey with a parent or guardian prior to participating, it was not feasible to track such conversations. Further to logistical issues in obtaining parental consent for online survey participation, more recent scholarly work suggests adolescents are capable of providing consent to social science anonymous surveys (50–52). Finally, adolescents who may be sexually active and/or are part of a sexuality/gender minority may be put at increased risk of harm should they be required to obtain parent/guardian consent which could lead to revealing a behavior/identity which the adult does not approve of resulting in harm (e.g., physical/verbal abuse, homelessness). The Ethics committee, for these reasons, approved a waiver of parental consent.

### Risks/Safeguards

There was a low risk of potential psychological or emotional stress for participants who were asked about their sexual experiences, particularly the unwanted ones. Reflection on the answers given may have caused minor distress. Given recent unwelcome media attention, there was an identified potential risk to investigators of the project including harassment leading to psychological, emotional, or social harm. While predicted to be minimal, recent media coverage of other youth-oriented projects has shown this to be a possibility (53).

Every page of the survey included web and phone links to Kids HelpLine and Lifeline where participants could engage with services to address any distress experienced in taking the survey. Additionally, links and phone numbers for Kids Help Line and LifeLine were provided at the end of the survey as well as in the resources section of the survey website and on the PIS. To protect the well-being of investigators, only the Chief Investigator was listed as the contact in the PIS. A university business email was used instead of the CI's email, and where appropriate, the CI screened incoming calls during data collection. The research team had pre-arranged support from the university media team to ensure appropriate responses to queries suspected of leading to press coverage. No phone calls were received during or after data collection and only two e-mails were received, one commenting on a lack of available options for persons identifying as asexual and one inquiring about the validity of the survey (e.g., was it a true academic study).

### Anticipated Results

Over the past 25 years, Australia has periodically conducted surveillance research on adolescent knowledge, behaviors, and more recently technology practices and educational experiences related to sexual health and well-being. Data from the 6th National Survey of Secondary Students and Adolescent Sexual Health adds to the robust history of knowledge of adolescent sexual health in Australia. Policymakers continue to utilize the information to inform national strategic priorities to address sexual health which in turn inform public health and health education responses to the latest trends in knowledge, behavior, and education. The research team anticipates continued rates of high impact resulting from the publication of descriptive data in a comprehensive report and subsequent dissemination through peer-reviewed publications, academic and community presentations, and further analyses to support public health and health education sectors in addressing adolescent sexual health and well-being.

### Participants

The 6th National Survey of Secondary Student and Adolescent Sexual Health was completed by 8,263 young people aged 14–18 living in Australia. Given the length of the survey, participants had to complete the knowledge and behavior sections of the survey, which contained the variables of primary interest to the funder, to remain in the final sample. The survey was started by 25,069 participants with 8,400 completed through the behavior section of the survey. Of those, 6,269 fully completed the survey. Data cleaning required the removal of 137 cases due to pranksters, usefulness of responses, and speeding (e.g., providing the same answer to series of questions, almost exclusive “don't know” or “prefer not to answer” responses or unreasonably quick completion time). Completion rate of the survey was 33.5%. Participants entering the draw for 1 of 20 $100 gift cards comprised 46.3% (*n* = 3,828) of the final sample.

The vast majority of respondents learned of the survey from the Facebook advertisement (*n* = 8,085, 97.8%) and completed the online survey via a mobile device (*n* = 7,256, 87.8%). The average completion time was 23.4 min (*SD* 8.04; due to the ability to stop and resume the survey within a 24 h period, such pauses were included in the tracked completion time; average time to complete was based on all responses taking <60 min; *n* = 7,893, 95.5%).

The sociodemographic characteristics of participants are shown in Table 1. Participants were relatively evenly split by traditional gender markers with slightly more females (*n* = 4,377, 53.0%). Younger participants (age 14–15) had lower levels of representation (*n* = 2,046, 24.7%) and a majority of participants were in Year 11 and 12 (*n* = 4,695, 56.8%). Government schools formed the largest education system represented in the survey (*n* = 3,645, 52.4%). The two most populous states, New South Wales and Victoria, also represented the majority of participants (*n* = 4,558, 55.2%). Across the primary and secondary strata used to determine minimum quotas based on census data, the sample of year 10, 11, and 12 students was well-distributed considering oversampling goals (see Table 2), with most unweighted strata within 1% of census levels and none >5% difference.

**Table 1:** Characteristics of participants (*N* = 8,263).

	***n***	**%**
**Age [mean (range)]**	16.26	
	(range 14–18)	
**Gender**		
Female	4,377	53.0
Male	3,685	44.6
Other	201	2.4
**Year in School**		
Year 9	605	7.3
Year 10	1,632	19.8
Year 11	2,387	28.9
Year 12	2,308	27.9
Not in School	1,219	14.8
Unspecified	112	1.4
**School Type (excludes not in school and unspecified)**		
Government	3,645	52.4
Catholic	1,676	24.1
Other Non-government	1,312	18.9
Not Sure	325	4.7
**State/Territory**		
Australian Capital Territory (ACT)	188	2.3
New South Wales (NSW)	2,264	27.4
Northern Territory (NT)	145	1.8
Queensland (QLD)	1,630	19.7
South Australia (SA)	688	8.3
Tasmania (TAS)	306	3.7
Victoria (VIC)	2,294	27.8
Western Australia (WA)	748	9.1
**Residence (defined by ABS remoteness, based on post-code)**		
Major cities of Australia	5787	70.0
Inner regional Australia	1,319	16.0
Outer regional Australia	441	5.3
Remote Australia	40	0.5
Very remote Australia	13	0.2
Unspecified	663	8.0
**Aboriginal or Torres Strait Islander**	339	4.2
(excludes “unspecified” *n* = 192)		
**Born in Australia**	7,450	90.7
(excludes “unspecified” *n* = 46)		
**Mother Born in Australia**	6,249	78.7
(excludes unspecified *n* = 324)		
**Father Born in Australia**	6,016	76.5
(excludes unspecified *n* = 396)		
**English Primary Language Spoken at Home**	7,853	95.4
(excludes unspecified *n* = 31)		
**Religion**		
Catholic	1,455	17.6
Anglican (Church of England)	454	5.5
Uniting Church	118	1.4
Presbyterian	83	1.0
Buddhism	67	0.8
Islam	48	0.6
Greek Orthodox	105	1.3
Baptist	114	1.4
Hinduism	23	0.3
Judaism	40	0.5
Other Christian religion	333	4.0
Other Non-Christian religion	119	1.4
No religion	5,036	60.9
Prefer Not to Answer	268	3.2
**Self-identified Sexual Orientation**		
Heterosexual or straight	5,959	73.0
Gay or lesbian	417	5.1
Bisexual	1,317	16.1
Not Sure	421	5.2
Prefer Not to Answer	51	0.6
(excludes unspecified *n* = 98)		
**Self-identified Gender Identity (check all that apply; % to total)**		
Male	3,669	44.9
Female	4,352	53.3
Transmale	73	0.9
Transfemale	9	0.1
Genderqueer/Gender Non-conforming	98	1.2
Another identity	28	0.3

**Table 2:** Sample comparison to census data by strata.

**School type**	**Gender**	**Year in School**	2016 Census Projections^*^	**Survey**	**Difference (%)**
			**% to Total**	**% to Total**	
Government	Male	Year 10	10.1%	5.7%	−4.4
		Year 11	9.5%	8.4%	−1.0
		Year 12	10.5%	8.5%	−2.1
	Female	Year 10	9.6%	8.8%	−0.7
		Year 11	8.8%	12.2%	3.4
		Year 12	9.9%	10.4%	0.5
Catholic	Male	Year 10	3.9%	2.6%	−1.3
		Year 11	3.8%	4.0%	0.2
		Year 12	3.9%	4.6%	0.7
	Female	Year 10	3.9%	3.9%	0.0
		Year 11	3.6%	5.7%	2.1
		Year 12	3.8%	4.8%	1.0
Other Non-government	Male	Year 10	3.1%	2.3%	−0.8
		Year 11	3.0%	3.8%	0.8
		Year 12	3.2%	4.4%	1.2
	Female	Year 10	3.1%	2.1%	−1.1
		Year 11	3.0%	3.7%	0.7
		Year 12	3.2%	4.1%	0.9
	**State/Territory**					
		ACT	1.8%	2.3%	0.4
		NSW	32.0%	28.3%	−3.7
		NT	1.1%	1.6%	0.5
		QLD	19.7%	17.7%	−2.0
		SA	7.1%	8.4%	1.4
		TAS	2.3%	3.9%	1.5
		VIC	25.0%	28.9%	3.9
		WA	10.9%	8.9%	−2.0

* *Projections based on a translation of 2016 data to 2018 projections for the year in school (e.g., year 8 in 2016 projected to be in year 10 in 2018); assumes no changes in school type or gender and 100% retention rates*.

Aboriginal and Torres Strait Islander youth representation in the survey (*n* = 399, 4.2%) was sufficiently oversampled in comparison to the overall national population level of 2.8% (54). Similarly, while a majority identified as heterosexual (*n* = 5,959, 73.0%), a substantial minority did not, suggesting a generous oversample based on international estimates of 2% of the population identifying as lesbian, gay or bisexual in the UK (55) and 3% identifying as gay, lesbian or “other” in Australia (56).

## Discussion

The 2018 National Survey of Secondary Students and Adolescent Sexual Health provides updated surveillance data on the knowledge, behaviors and educational experiences of Australian youth. The 6th installment of the Secondary Student Survey builds on the impacts of previous versions of the survey by informing the work of health professionals, teachers, youth workers, service planners, and policymakers. For example, a priority area for action in the national STI strategy (1) includes education and prevention, “including supporting improved sexual health education in schools…to improve knowledge and awareness of healthy relationships and STI and reduce risk behaviours associated with the transmission of STI.” (p. 25). The Secondary Student Survey provides vital data on current levels of STI knowledge, current levels of risk behaviors and current educational experiences need to develop responses to this priority area found in the related Key Area for Action to “implement a national STI education initiative…to improve…understanding of STI, improve knowledge of risk behaviours and safer sex practices.” (p. 26). Findings will indicate what knowledge is known (and lacking), which behaviors young people are (and are not) engaging in and the types of sexual health education experiences they desire.

Beyond impacts in the field, the 2018 survey will, through academic channels (e.g., peer-reviewed publications, conference presentations), expand and update the scope of knowledge into young people's sexual health and well-being. The survey is one of very few world-wide to provide ongoing robust in-depth research into adolescent sex-related knowledge, behaviors and educational experiences. The United States has a long history of work in this space, though national data are limited to basic information into sexual behaviors [e.g., Add Health, YRBS; (57, 58)] with the exception of the more in-depth national work on behaviors from Indiana University [NSSHB; (59)]. Similarly, research in the UK and Europe [e.g., HBSC (60), NatSAL (61)] are limited to basic data on behaviors and do not include knowledge or educational experiences. No other national or international surveys examine adolescent knowledge on STIs and HIV or the informal and formal experiences of sexual health education.

The Australian survey provides a unique contribution to the field by covering, not only behavior or knowledge or educational experiences, but by covering all of them in one survey. Combined with the large, diverse sample obtained, the potential for examining, in-depth, a number of adolescent sexual health and well-being issues, will likely generate many new contributions to understanding the relationships between knowledge, education and behavior across a diverse sample of adolescents. In particular, the constructs comprehensively measured (knowledge, behavior, and educational experiences) in the survey allow analyses that will contribute to the evaluation and development of theoretical perspectives in the field.

The cross-sectional repeated nature of the survey provides a second equally important contribution to the field. Across six waves over 25 years, the survey has asked the same fundamental questions, often using identical wording. This consistency provides the possibility of analyzing across cohorts' changes in knowledge, behavior and informal educational experiences. Mapping these experiences to policy, technological, medical, social and cultural shifts may provide a window into how adolescent sexual health and behavior has and has not changed since 1992. Understanding what has and has not changed, and under which contexts, may help to inform a broader understanding of the intricate interactions at a systems level that impact on adolescent sexual health and well-being (62, 63).

Finally, the innovative methodology of the survey, namely changes in the recruitment and sampling procedures from previous iterations, may help to inform future national (and international) surveys with adolescent populations on sensitive topics. The increasingly structured curriculum and expected measurable learning outcomes (64, 65) leaves less time in schools to conduct traditional two-tier random cluster sampling procedures considered the “gold standard” in school-aged population research (66, 67), a standard further complicated by increasingly limited funding for such research endeavors. The approach used documents obtaining national samples of young people recruited online to participate in sexual health research as not only feasible but achievable.

### Strengths and Limitations

The primary limitation of the survey is the potential for generalizability. First, despite using a very widely used platform for recruitment, not every young person in Australia is on Facebook, nor would all users have seen the advertisements. Similar to many other sexual health studies, the Secondary Student Survey likely suffered a selection bias (68, 69). Other limitations include only being accessible to participants who could read and respond to an English language survey and had access to and knowledge of how to use an internet-enabled device. The size and diversity of the sample, however, provide a very good snapshot of the sexual health knowledge, behaviors, and educational experiences of Australian adolescents, which would be hard to assess otherwise.

### Benefits

The survey findings can be used widely throughout Australia, guiding the work of health professionals, teachers, youth workers, service planners, and policymakers. Survey results can be used to inform educational policy and sexual health programs, to improve the relevance of sexual health resources available to teachers, and by health departments to plan interventions for young people in Australia. Results can be published in reports that are made available to schools and education authorities, as well as published in academic journals and presented at scientific conferences. Results will inform progress on the National Sexually Transmissible Infections Strategy and state and territory policies and plans for supporting the sexual health of young people.

## Data Availability

The datasets for this manuscript are not publicly available because the protocol approved by the Human Ethics Committee did not explicitly provide participants notice of the datasets being publicly available in the participant information sheet and subsequent agreement to participate process. Requests to access the datasets should be directed to Associate Professor CF (c.fisher2@latrobe.edu.au).

## Dissemination

Research products arising from the survey will include a national public report of descriptive findings, presentations at academic and sector conferences and professional development workshops, and peer-reviewed papers.

## Ethics Statement

Recommendations of the Australian National Health and Medical Research Council National Statement on Ethical Conduct in Human Ethical Conduct was followed in the development and implementation of the study. La Trobe University Human Ethics Committee provided the approval of all protocols (HEC 18030). All participants of the anonymous survey provided assent to participate.

## Author Contributions

CF and GM conducted the data analysis. CF and JL prepared the first draft of the manuscript. CF, GM, PE, LK, RB, GB, and JL contributed to the study design, development of the survey instrument and recruitment. CF, GM, PE, LK, RB, GB, AW, and JL contributed to further development of and editing the manuscript.

### Conflict of Interest Statement

The authors declare that the research was conducted in the absence of any commercial or financial relationships that could be construed as a potential conflict of interest.

## References

[1] Australian Government Department of Health Fourth National Sexually Transmissible Infections Strategy 2018-2022. Canberra, ACT (2018).

[2] Australian Government Department of Health Eighth National HIV Strategy 2018-2022. Canberra, ACT (2018).

[3] The Kirby Institute HIV, Viral Hepatitis and Sexually Transmissible Infections in Australia: Annual Surveillance Report 2018. Sydney, NSW (2018).

[4] Becker MH The health belief model and personal health behavior. Health Educ Monogr. (1974) 2:324–473. 10.1177/109019817400200407

[5] Ajzen I From intentions to actions: a theory of planned behavior. In: Kuhland J Beckman J editors. Action-Control: From Cognitions to Behavior. Heidelberg: Springer (1985). p. 11–39. 10.1007/978-3-642-69746-3_2

[6] Fishbein M Ajzen I Belief, Attitude, Intention and Behavior: An Introduction to Theory and Research. Reading, MA: Addison-Wesley (1975).

[7] Bandura A Social Foundations of Thought and Action: A Social Cognitive Theory. Englewood Cliffs, NJ: Prentice-Hall (1986).

[8] Prochaska JO Velicer WF. The transtheoretical model of health behavior change. Am J Health Promot. (1997) 12:38–48. 10.4278/0890-1171-12.1.38 10170434

[9] Hiltabiddle SJ. Adolescent condom use, the health belief model, and the prevention of sexually transmitted disease. J Obst Gynecol Neonatal Nurs. (1996) 25:61–6. 10.1111/j.1552-6909.1996.tb02514.x 8627404

[10] Hall KS. The health belief model can guide modern contraceptive behavior research and practice. J Midwifery Womens Health. (2011) 57:74–81. 10.1111/j.1542-2011.2011.00110.x 22251916 PMC3790325

[11] Reece M Herbenick D Schick V Sanders SA Dodge B Fortenberry JD. Background and considerations on the National Survey of Sexual Health and Behavior (NSSHB) from the investigators. J Sex Med. (2010) 7(Suppl. 5):243–5. 10.1111/j.1743-6109.2010.02038.x 21029382

[12] Rotermann M. Sexual behaviour and condom use of 15- to 24-year-olds in 2003 and 2009/2010. Health Rep. (2012) 23:41–5. 22590804

[13] Schluter M Baeza A Dressler G Frank K Groeneveld J Jarger W A framework for mapping and comparing behavioural theories in models of social-ecological systems. Ecol Econ. (2017) 131:21–35. 10.1016/j.ecolecon.2016.08.008

[14] Breuner CC Mattson G Health CoPAoCaF. Sexuality education for children and adolescents. Pediatrics. (2016) 138:e20161348. 10.1542/peds.2016-1348 27432844

[15] Australian Bureau of Statistics Australian Demographic Statistics (2018).

[16] Australian Bureau of Statistics State and Territory Compisition of Country of Birth. (2018). Available online at: https://www.abs.gov.au/ausstats/abs@.nsf/Latestproducts/3412.0Main%20Features42016-17?opendocument&tabname=Summary&prodno=3412.0&issue=2016-17&num=&view= (accessed May 1, 2019).

[17] Dunne MP Donald M Lucke J Nilsson R Ballard R Raphael B. Age-related increase in sexual behaviours and decrease in regular condom use among adolescents in Australia. Int J STD AIDS. (1994) 5:41–7. 10.1177/095646249400500110 8142527

[18] Dunne MP Edwards R Lucke J Donald M Raphael B. Religiosity, sexual intercourse and condom use among university students. Aust J Public Health. (1994) 18:339–41. 10.1111/j.1753-6405.1994.tb00257.x 7841268

[19] Mitchell A Patrick K Heywood W Blackman P Pitts M 5th National Survey of Australian Secondary Students and Sexual Health 2013. Melbourne, VIC: La Trobe University (2014).

[20] Mendes R Plaza V Wallerstein N. Sustainability and power in health promotion: community-based participatory research in a reproductive health policy case study in New Mexico. Global Health Promotion. (2016) 23:61–74. 10.1177/1757975914550255 25432963

[21] Australian Bureau of Statistics 1270.0.55.005 - Australian Statistical Gepgraphy Standard (ASGS): Volume 5 - Remoteness Structure, July 2016: Correspondence, 2017 Postcode to 2016 Remoteness Area. Canberra, ACT: Australian Bureau of Statistics (2018). Available online at: http://www.abs.gov.au/AUSSTATS/abs@.nsf/DetailsPage/1270.0.55.005July%202016?OpenDocument (accessed May 1, 2019).

[22] The GenIUSS Group Best Practices for Asking Questions to Identify Transgender and Other Gender Minority Respondents on Population-Based Surveys. Los Angeles, CA: The Williams Institute (2014).

[23] Mahat G Scoloveno MA HIV peer education: relationahips between adolescents' HIV/AIDS knowledge and self-efficacy. J HIV AIDS Soc Serv. (2010) 9:371–84. 10.1080/15381501.2010.525479

[24] Swenson RR Rizzo CJ Brown LK Vanable PA Carey MP Valois RF. HIV knowledge and its contribution to sexual health behaviors of low-income african American adolescents. J Natl Med Assoc. (2010) 102:1173–82. 10.1016/S0027-9684(15)30772-0 21287898 PMC3095017

[25] Tan X Jingju P Zhou D Wang C Xie C. HIV/AIDS knowledge, attitudes and behaviors assessment of Chinese students: a questionnaire study. Int J Environ Res Public Health. (2017) 4:248–53. 10.3390/ijerph2007030009 17911665 PMC3731642

[26] Davey-Rothwell MA Tobin K Yang C Sun CJ Latkin CA. Results of a randomized controlled trial of a peer mentor HIV/STI prevention intervention for women over an 18 month follow-up. AIDS Behav. (2011) 15:1654–63. 10.1007/s10461-011-9943-9 21468659 PMC3158274

[27] Australian Government Department of Health Fifth National Hepatits C Strategy 2018-2022. Canberra, ACT (2018).

[28] Australian Government Department of Health Third National Hepatits B Strategy 2018-2022. Canberra, ACT (2018).

[29] My Healthy Communities Australian Institute of Health and Welfare HPV Immunisation Rates 2015-16. (2019). Available online at: https://myhealthycommunities.gov.au/our-reports/HPV-rates/march-2018 (accessed May 1, 2019).

[30] Green EC Murphy E Health belief model. In: Cockerham WC Dingwall R Quah S editors. The Wiley Blackwell Encyclopedia of Health, Illness, Behavior, and Society. Hoboken, NJ: Wiley-Blackwell (2014). p. 766–9. 10.1002/9781118410868.wbehibs410

[31] Wang K Brown K Shen SY Tucker J. Social network-based interventions to promote condom use: a systematic review. AIDS Behav. (2011) 15:1298–308. 10.1007/s10461-011-0020-1 21811843 PMC3220661

[32] Peterson Z Muehlenhard C. What is sex and why does it matter? A motivational approach to exploring individuals' definitions of sex. J Sex Res. (2007) 44:256–68. 10.1080/00224490701443932 17879169

[33] Sewell KK Strassberg DS. How do heterosexual undergraduate students define having sex? A new approach to an old question. J Sex Res. (2015) 52:507–16. 10.1080/00224499.2014.888389 24742052

[34] Centers for Disease Control and Prevention HIV Transmission. (2018). Available online at: https://www.poz.com/basics/hiv-basics/hiv-transmission-risks (accessed May 1, 2019).

[35] Centers for Disease Control and Prevention Sexually Transmitted Diseases (STDs). (2019). Available online at: https://www.cdc.gov/std/default.htm (accessed May 1, 2019).

[36] Herold ES Goodwin MS Reasons given by female virgins for not having premarital intercourse. J Sch Health. (1981) 51:496–500. 10.1111/j.1746-1561.1981.tb05338.x 6912338

[37] Miller BC Norton MC Fan XT Christopherson CR Pubertal development, parental communication, and sexual values in relation to adolescent sexual behaviors. J Early Adolesc. (1998) 18:27–52. 10.1177/0272431698018001002

[38] Sprecher S Regan PC College virgins: how men and women perceive their sexual status. J Sex Res. (1996) 33:3–15. 10.1080/00224499609551810

[39] DiClemente RJ Swartzendruber AL Brown JL. Improving the validity of self-reported sexual behavior: no easy answers. Sex Transm Dis. (2013) 40:111–2. 10.1097/OLQ.0b013e3182838474 23321991 PMC3846295

[40] Sensis Yellow social media report 2018. Part One – Consumers (2018).

[41] Patrick K Heywood W Pitts MK Mitchell A. Demographic and behavioural correlates of six sexting behaviours among Australian secondary school students. Sex Health. (2015) 12:480–7. 10.1071/SH15004 26277625

[42] Szucs E Sex talk online: sexual self-construction in adolescent internet spaces. Girlhood studies. Interdisc J. (2013) 6:117 10.3167/ghs.2013.060109

[43] United Nations Organization for Education Science and Culture International Technical Guidance on Sexuality Education: An evidence-Informed Approach for Schools, Teachers and Health Educators. Paris: UNESCO (2009).

[44] Lindsay J Smith A Rosenthal D Secondary Students, HIV/AIDS and Sexual Health. Centre for the Study of Sexually Tranmissible Diseases Melbourne, VIC (1997).

[45] Dunne M Donald M Lucke J Nilsson R Raphael B 1992 HIV Risk and Sexual Behaviour Survey in Australian Secondary Schools. Canberra, ACT: National Centre for HIV Social Research (1993).

[46] Smith A Agius P Dyson S Mitchell A Pitts M Seconday Students and Sexual Health 2002: Results of the 3rd National Survey of Australian Secondary Students, HIV/AIDS and Sexual Health. Melbourne, VIC: Australian Research Centre in Sex, Health & Society (2003).

[47] Smith A Agius P Mitchell A Barrett C Pitts M Secondary Students and Sexual Health 2008: Results of the 4th National Survey of Australian Secondary Students. Melbourne, VIC: La Trobe University (2009).

[48] Panacek EA Thompson CB. Sampling methods: selecting your subjects. Air Med J. (2007) 26:75–8. 10.1016/j.amj.2007.01.001 17346642

[49] The National Health and Medical Research Council tARC Universities Australia National Statement on Ethical Conduct in Human Research 2007 (Updated 2018). Canberra, ACT: National Health and Medical Research Council (2018).

[50] Flicker S Guta A. Ethical approaches to adolescent participation in sexual health research. J Adolesc Health. (2008) 42:3–10. 10.1016/j.jadohealth.2007.07.017 18155024

[51] Kuyper L de Wit J Adam P Woertman L. Doing more good than harm? The effects of participation in sex research on young people in the Netherlands. Arch Sex Behav. (2012) 41:497–506. 10.1007/s10508-011-9780-y 21681692

[52] Shaw T Cross D Thomas LT Zubrick SR Bias in student survey findings from active parental consent procedures. Br Educ Res J. (2015) 41:229–43. 10.1002/berj.3137

[53] aaaa Personal Responsibility and Work Opportunity Reconciliation Act of 1996. Pub. L. 104–93, 110 Stat. 2105 (August 22, 1996).

[54] Australian Bureau of Statistics Census of Population and Housing: Characteristics of Aboriginal and Torres Straight Islander Australians. (2016). Available online at: https://www.abs.gov.au/ausstats/abs@.nsf/mf/2076.0 (accessed May 1, 2019).

[55] United Kingdom Government Office for National Statistics Sexual Orientation, UK: 2017. (2017). Available online at: https://www.ons.gov.uk/peoplepopulationandcommunity/culturalidentity/sexuality/bulletins/sexualidentityuk/2017 (accessed May 1, 2019).

[56] Australian Bureau of Statistics 4159.0 - General Social Survey: Summary Results, Australia, 2014. (2015).

[57] Centers for Disease Control and Prevention Youth Risk Behavior Survey Data (2017).

[58] Harris KM Udry JR National Longitudinal Study of Adolescent to Adult Health (Add Health), 1994-2008 [Public Use]. Ann Arbor, MI: Carolina Population Center, University of North Carolina-Chapel Hill [distributor], Inter-university Consortium for Political and Social Research [distributor] (2018). 10.3886/ICPSR21600.v21

[59] Herbenick D Reece M Schick V Sanders SA Dodge B Fortenberry JD. Sexual behavior in the United States: results from a national probability sample of men and women ages 14–94. J Sex Med. (2010) 7(Suppl. 5):255–65. 10.1111/j.1743-6109.2010.02012.x 21029383

[60] Young H Kolto A Reis M Saewyc EM Moreau N Burke L. Sexual Health questions included in the Health Behaviour in School-aged Children (HBSC) study: an international methodological pilot investigation. BMC Med Res Methodol. (2016) 16:169. 10.1186/s12874-016-0270-8 27919233 PMC5139025

[61] Mitchell KR Mercer CH Ploubidis GB Jones KG Datta J Field N. Sexual function in Britain: findings from the third National Survey of Sexual Attitudes and Lifestyles (Natsal-3). Lancet. (2013) 382:1817–29. 10.1016/S0140-6736(13)62366-1 24286787 PMC3898902

[62] Chen AC-C Thompson EA Morrison-Beedy D. Multi-system influences on adolescent risky sexual behavior. Res Nurs Health. (2010) 33:512–27. 10.1002/nur.20409 21053385

[63] Viner RM Ozer EM Denny S Marmot M Resnick M Fatusi A. Adolescence and the social determinants of health. Lancet. (2012) 379:1641–52. 10.1016/S0140-6736(12)60149-4 22538179

[64] Ambrosio J Changing the subject: neoliberalism and Accountability in public education. Educ Stud. (2013) 49:316–33. 10.1080/00131946.2013.783835

[65] Brancaleone D O'Brien S Educational commodification and the (Economic) sign value of learning outcomes. Br J Sociol Educ. (2011) 32:501–19. 10.1080/01425692.2011.578435

[66] Hedges LV Effect sizes in three-level cluster-randomized experiments. J Educ and Behav Statist. (2011) 36:346–80. 10.3102/1076998610376617

[67] Planty M Carlson D Understanding Education Indicators: A Practical Primer for Research and Policy. New York, NY: Teachers College Press (2010).

[68] Bethlehem J Selection bias in web surveys. Int Statist Rev. (2010) 78:161–88. 10.1111/j.1751-5823.2010.00112.x

[69] Janssens ACJW Kraft P. Research conducted using data obtained through online communities: Ethic Implic Methodol Limit. (2012) 9:e1001328. 10.1371/journal.pmed.1001328 23109913 PMC3479089

