# Supplementary material for: Study Protocol: 6th National Survey of Australian Secondary Students and Adolescent Sexual Health, 2018
Source: Front Public Health. 2019 Aug 22;7:217. doi: 10.3389/fpubh.2019.00217 (PMC6714884; doi:10.3389/fpubh.2019.00217)
Supplement: Supplementary file 1 [file Table_1.DOCX]

Appendix

6^th^ National Survey of Secondary Students and Adolescent Sexual Health, 2018

Survey Instrument

- Can only choose one (1) option
- Can check more than one option

____ Follow-up question to provide fill-in-the-blank response

Number in parentheses (#) next to each response represents the assigned value to the response for skip pattern purposes.

Survey items are arranged by domain; this does not reflect the exact order of questions as presented to participants. However, generally the survey first covered knowledge, then behaviours and ended with educational experiences.

**QUALIFICATION QUESTIONS**

**1** In which Australian state/territory do you live?

- Australian Capital Territory (1)
- New South Wales (2)
- Northern Territory (3)
- Queensland (4)
- South Australia (5)
- Tasmania (6)
- Victoria (7)
- Western Australia (8)
- I do not live in Australia (9)

**2** What is your age?

- Under 14 years old (1)
- 14 (2)
- 15 (3)
- 16 (4)
- 17 (5)
- 18 (6)
- 19 years or older (7)

**3** How did you hear about this survey?

- Ad on my Facebook page (1)
- Ad sent to me by a friend through Facebook (2)
- Link sent to me by a friend (3)
- Link sent to me by a teacher (4)
- Link sent to me by a sibling (5)
- Link sent to me by a parent/guardian/step-parent (6)
- Found it through an online search (7)
- Other _____________________ (8)
- Not Sure (9)

Disqualified from participation if **1 = 9** and/or **2 = 1** or **2 = 7**

**A: SOCIO-DEMOGRAPHICS**

**A1** Are you?

- Male (1)
- Female (2)
- Other_________ (3)
- Prefer Not To Answer (4)

**A2** Please select your birth month:

- January (1)
- February (2)
- March (3)
- April (4)
- May (5)
- June (6)
- July (7)
- August (8)
- September (9)
- October (10)
- November (11)
- December (12)
- Prefer Not To Answer (13)

**A3** Please select your birth year:

- 1999 (1)
- 2000 (2)
- 2001 (3)
- 2002 (4)
- 2003 (5)
- 2004 (6)
- 2005 (7)
- Prefer Not To Answer (8)

**A4** Which year are you in at school?

- Year 9 (1)
- Year 10 (2)
- Year 11 (3)
- Year 12 (4)
- I am not in school (5)
- Prefer Not To Answer (6)

***Skip to A7 if A4 = 5***

**A5** What type of school do you go to?

- Government (1)
- Catholic (2)
- Other non-government (3)
- Not Sure (4)
- Prefer Not To Answer (5)

**A6** Which best describes your school?

- All boys school (1)
- All girls school (2)
- Mixed gender school (3)
- Home school (4)
- Prefer Not To Answer (5)

**A7** What is the post code of your home address? (Type "prefer not to answer" or "don't know" if appropriate): _________________

**A8** Were you born in Australia?

- Yes (1)
- No (2)
- Not Sure (3)
- Prefer Not To Answer (4)

***Skip to A9 if A8*** $\boldsymbol{\neq}$ ***2***

**A8a** In which country were you born?

- Not Sure (196)
- Prefer Not To Answer (197)
- Afghanistan (1)
- Albania (2)
- Algeria (3)
- Andorra (4)
- Angola (5)
- Antigua and Barbuda (6)
- Argentina (7)
- Armenia (8)
- Austria (10)
- Azerbaijan (11)
- Bahamas (12)
- Bahrain (13)
- Bangladesh (14)
- Barbados (15)
- Belarus (16)
- Belgium (17)
- Belize (18)
- Benin (19)
- Bhutan (20)
- Bolivia (21)
- Bosnia and Herzegovina (22)
- Botswana (23)
- Brazil (24)
- Brunei Darussalam (25)
- Bulgaria (26)
- Burkina Faso (27)
- Burundi (28)
- Cambodia (29)
- Cameroon (30)
- Canada (31)
- Cape Verde (32)
- Central African Republic (33)
- Chad (34)
- Chile (35)
- China (36)
- Colombia (37)
- Comoros (38)
- Congo, Republic of the (39)
- Costa Rica (40)
- Côte d'Ivoire (41)
- Croatia (42)
- Cuba (43)
- Cyprus (44)
- Czech Republic (45)
- Democratic People's Republic of Korea (46)
- Democratic Republic of the Congo (47)
- Denmark (48)
- Djibouti (49)
- Dominica (50)
- Dominican Republic (51)
- Ecuador (52)
- Egypt (53)
- El Salvador (54)
- Equatorial Guinea (55)
- Eritrea (56)
- Estonia (57)
- Ethiopia (58)
- Fiji (59)
- Finland (60)
- France (61)
- Gabon (62)
- Gambia (63)
- Georgia (64)
- Germany (65)
- Ghana (66)
- Greece (67)
- Grenada (68)
- Guatemala (69)
- Guinea (70)
- Guinea-Bissau (71)
- Guyana (72)
- Haiti (73)
- Honduras (74)
- Hong Kong (S.A.R.) (75)
- Hungary (76)
- Iceland (77)
- India (78)
- Indonesia (79)
- Iran, Islamic Republic of (80)
- Iraq (81)
- Ireland (82)
- Israel (83)
- Italy (84)
- Jamaica (85)
- Japan (86)
- Jordan (87)
- Kazakhstan (88)
- Kenya (89)
- Kiribati (90)
- Kuwait (91)
- Kyrgyzstan (92)
- Lao People's Democratic Republic (93)
- Latvia (94)
- Lebanon (95)
- Lesotho (96)
- Liberia (97)
- Libyan Arab Jamahiriya (98)
- Liechtenstein (99)
- Lithuania (100)
- Luxembourg (101)
- Madagascar (102)
- Malawi (103)
- Malaysia (104)
- Maldives (105)
- Mali (106)
- Malta (107)
- Marshall Islands (108)
- Mauritania (109)
- Mauritius (110)
- Mexico (111)
- Micronesia, Federated States of (112)
- Monaco (113)
- Mongolia (114)
- Montenegro (115)
- Morocco (116)
- Mozambique (117)
- Myanmar (118)
- Namibia (119)
- Naur (120)
- Nepal (121)
- Netherlands (122)
- New Zealand (123)
- Nicaragua (124)
- Niger (125)
- Nigeria (126)
- North Korea (127)
- Norway (128)
- Oman (129)
- Pakistan (130)
- Palau (131)
- Panama (132)
- Papua New Guinea (133)
- Paraguay (134)
- Peru (135)
- Philippines (136)
- Poland (137)
- Portugal (138)
- Qatar (139)
- Republic of Korea (140)
- Republic of Moldova (141)
- Romania (142)
- Russian Federation (143)
- Rwanda (144)
- Saint Kitts and Nevis (145)
- Saint Lucia (146)
- Saint Vincent and the Grenadines (147)
- Samoa (148)
- San Marino (149)
- Sao Tome and Principe (150)
- Saudi Arabia (151)
- Senegal (152)
- Serbia (153)
- Seychelles (154)
- Sierra Leone (155)
- Singapore (156)
- Slovakia (157)
- Slovenia (158)
- Solomon Islands (159)
- Somalia (160)
- South Africa (161)
- South Korea (162)
- Spain (163)
- Sri Lanka (164)
- Sudan (165)
- Suriname (166)
- Swaziland (167)
- Sweden (168)
- Switzerland (169)
- Syrian Arab Republic (170)
- Tajikistan (171)
- Thailand (172)
- The former Yugoslav Republic of Macedonia (173)
- Timor-Leste (174)
- Togo (175)
- Tonga (176)
- Trinidad and Tobago (177)
- Tunisia (178)
- Turkey (179)
- Turkmenistan (180)
- Tuvalu (181)
- Uganda (182)
- Ukraine (183)
- United Arab Emirates (184)
- United Kingdom of Great Britain and Northern Ireland (185)
- United Republic of Tanzania (186)
- United States of America (187)
- Uruguay (188)
- Uzbekistan (189)
- Vanuatu (190)
- Venezuela, Bolivarian Republic of (191)
- Viet Nam (192)
- Yemen (193)
- Zambia (580)
- Zimbabwe (1357)
- Other (198)

**A8b** In what year did you arrive in Australia to live here for one year or more?

- 1999 (1)
- 2000 (2)
- 2001 (3)
- 2002 (4)
- 2003 (5)
- 2004 (6)
- 2005 (7)
- 2006 (8)
- 2007 (9)
- 2008 (10)
- 2009 (11)
- 2010 (12)
- 2011 (13)
- 2012 (14)
- 2013 (15)
- 2014 (16)
- 2015 (17)
- 2016 (18)
- 2017 (19)
- Been here less than a year (20)
- Prefer Not To Answer (21)

**A9** Are you of Aboriginal or Torres Strait Islander origin?

- No (1)
- Yes, Aboriginal (2)
- Yes, Torres Strait Islander (3)
- Yes, Aboriginal and Torres Strait Islander (4)
- Not Sure (5)
- Prefer Not To Answer (6)

**A10** In which country was your mother born?

(for options, see above list for A8a plus Australia toward top of list)

**A11** In which country was your father born?

(for options, see above list for A8a plus Australia toward top of list)

**A12** Is English the main language spoken at home?

- Yes (1)
- No (2)
- Prefer Not To Answer (3)

***Display A12a if A12 = 2***

**A12a** Please specify the main language spoken at home

- Australian Aboriginal language (1)
- Arabic (2)
- Cantonese (3)
- Greek (4)
- Hindi (5)
- Italian (6)
- Mandarin (7)
- Punjabi (8)
- Spanish (9)
- Tagalog (10)
- Vietnamese (11)
- Other______ (12)
- Prefer Not To Answer (13)

**A13** What religion do you follow?

- Catholic (1)
- Anglican (Church of England) (2)
- Uniting Church (3)
- Presbyterian (4)
- Buddhism (5)
- Islam (6)
- Greek Orthodox (7)
- Baptist (8)
- Hinduism (9)
- Judaism (10)
- Other Christian religion ______ (11)
- Other non-Christian religion __ (12)
- No religion (13)
- Prefer Not To Answer (14)

**A14** Do you consider yourself to be:

- Heterosexual or straight (1)
- Gay or lesbian (2)
- Bisexual (3)
- Not sure (4)
- Prefer Not To Answer (5)

**A15** People are different in their sexual attraction to other people. Which best describes your feelings? Are you:

- Only attracted to females (1)
- Mostly attracted to females (2)
- Equally attracted to females and males (3)
- Mostly attracted to males (4)
- Only attracted to males (5)
- Not sure (6)
- Prefer Not To Answer (7)

**A16** What sex were you assigned at birth, on your original birth certificate?

- Male (1)
- Female (2)
- Not sure (3)
- Prefer Not To Answer (4)

**A17** What is your current gender identity? Please check all that apply.

- Male (1)
- Female (2)
- Trans male/Trans man (3)
- Trans female/Trans woman (4)
- Genderqueer/Gender non-conforming (5)
- Different identity ____________ (6)
- Prefer Not To Answer (7)

**B: KNOWLEDGE**

***HIV Knowledge***

**B1** This section asks you what you know about HIV, or Human Immunodeficiency Virus, sometimes called the AIDS virus.

**B1a-k options (correct answer noted in italics)**

- Yes (1)
- No (2)
- I’m not sure (3)
- Prefer not to Answer (4)

B1a Could a person get HIV (the AIDS virus) by sharing a needle and syringe with someone when injecting drugs? *(Yes)*

B1b Could a woman get HIV (the AIDS virus) through having sex with a man? *(Yes)*

B1c If someone with HIV coughs or sneezes near other people, could they get the virus? *(No)*

B1d Could a man get HIV through having sex with a man? *(Yes)*

B1e Could a person get HIV from mosquitoes? *(No)*

B1f If a woman with HIV is pregnant, could her baby become infected with HIV? *(Yes)*

B1g Could a person get HIV by hugging someone who has it? *(No)*

B1h Does the pill (birth control) protect a woman from HIV infection? *(No)*

B1i Could a man get HIV through having sex with a woman? *(Yes)*

B1j If condoms are used during sex, does this help to protect people from getting HIV? *(Yes)*

B1k Could someone who looks very healthy pass on HIV infection? *(Yes)*

***STI Knowledge*B2** This question asks you what you know about hepatitis and sexually transmissible infections.

**B2a-s options (correct answer noted in italics)**

- True (1)
- False (2)
- Don’t know (3)
- Prefer not to Answer (4)

B2a Apart from HIV, all sexually transmissible infections can be cured (*False)*

B2b Chlamydia can lead to sterility among women *(True)*

B2c Chlamydia is a sexually transmissible infection that affects only women *(False)*

B2d Cold sores and genital herpes can be caused by the same virus *(True)*

B2e Genital warts can only be spread by intercourse *(False)*

B2f Gonorrhoea can be transmitted during oral sex *(True)*

B2g Hepatitis C can be transmitted by tattooing and body piercing *(True)*

B2h Hepatitis C has no long-term effects on your health *(False)*

B2i Hepatitis B can be transmitted sexually *(True)*

B2j Hepatitis C can be transmitted by sharing razors or toothbrushes *(True)*

B2k HIV only infects gay men and injecting drug users *(False)*

B2l It is possible to be vaccinated against hepatitis A *(True)*

B2m It is possible to be vaccinated against hepatitis B *(True)*

B2n It is possible to be vaccinated against hepatitis C *(False)*

B2o Once a person has caught genital herpes, then they will always have the virus *(True)*

B2p People who always use condoms are safe from all STIs *(False)*

B2q People who have injected drugs are not at risk for hepatitis C *(False)*

B2r Someone can have a sexually transmissible infection without any obvious symptoms *(True)*

B2s There is a cure for hepatitis C which is 90-95% effective *(True)*

**B3** This question asks you what you know about symptoms of sexually transmissible infections (STI).

**B3a-g options (correct answer noted in italics)**

- True (1)
- False (2)
- Don’t know (3)
- Prefer not to Answer (4)

B3a Discharge from the penis or vagina can be a symptom of an STI *(True)*

B3b Pain or discomfort when urinating can be a symptom of an STI *(True)*

B3c Muscular soreness in the thighs can be a symptom of an STI *(True)*

B3d Lumps and bumps in the genital area can be a symptom of an STI *(True)*

B3e Severe headache can be a symptom of an STI *(True)*

B3f Discoloured skin in the genital area can be a symptom of an STI *(True)*

B3g A rash in the genital area can be a symptom of an STI *(True)*

**B4** This question asks you what you know about HPV, also known as Human Papilloma Virus.

**B4a-o options (correct answer noted in italics)**

- Yes (1)
- No (2)
- I’m not sure (3)
- Prefer not to Answer (4)

B4a Have you heard of the HPV virus?

B4b HPV affects only or mainly men *(No)*

B4c HPV affects only or mainly women *(No)*

B4d HPV affects both men and women *(Yes)*

B4e HPV is the virus that causes genital warts *(Yes)*

B4f HPV causes cervical cancer in women *(Yes)*

B4g HPV causes cancers of the head and the throat *(Yes)*

B4h Using condoms when you have sex gives complete protection against HPV *(No)*

B4i You can tell if you have HPV *(No)*

B4j Being infected with HPV always leads to cervical cancer *(No)*

B4k Vaccinating young people against HPV would encourage them to become sexually active *(No)*

B4l The HPV vaccination won’t work if a person is already sexually active *(No)*

B4m The HPV vaccine gives you HPV *(No)*

B4n My GP can give me the HPV vaccine free of charge *(Yes)*

B4o If a woman has had the HPV vaccination she also needs to have regular cervical cancer tests *(Yes)*

**C: Behaviour**

***Perceived Susceptibility***

**C1** How likely do you think you are personally to get HIV infection?

- Never (1)
- Very unlikely (2)
- Unlikely (3)
- Likely (4)
- Very likely (5)
- Prefer Not To Answer

**C2** How likely do you think you are personally to get any STI?

- Never (1)
- Very unlikely (2)
- Unlikely (3)
- Likely (4)
- Very likely (5)
- Prefer Not To Answer (6)

**C3** How likely do you think you are personally to get hepatitis B?

- Never (1)
- Very unlikely (2)
- Unlikely (3)
- Likely (4)
- Very likely (5)
- Prefer Not To Answer (6)

**C4** How likely do you think you are personally to get hepatitis C?

- Never (1)
- Very unlikely (2)
- Unlikely (3)
- Likely (4)
- Very likely (5)
- Prefer Not To Answer (6)

***Protective Behaviours***

**C5** Have you been vaccinated against HPV, also called the cervical cancer vaccine?

- Yes (1)
- No (2)
- Don’t know (3)
- Prefer Not To Answer (4)

***Display this question if C5 = 1***

**C5a** How many doses of HPV vaccine have you received?

- 1 dose (1)
- 2 doses (2)
- 3 doses (3)
- 4 doses (4)
- Don’t remember (5)
- Prefer not to answer (6)

**C6** Have you been vaccinated against hepatitis A?

- Yes (1)
- No (2)
- Don’t know (3)
- Prefer Not To Answer (4)

**C7** Have you been vaccinated against hepatitis B?

- Yes (1)
- No (2)
- Don’t know (3)
- Prefer Not To Answer (4)

**C8** Have you been vaccinated against hepatitis C?

- Yes (1)
- No (2)
- Don’t know (3)
- Prefer Not To Answer (4)

**C9** Have you been diagnosed with hepatitis?

- Yes (1)
- No (2)
- Don’t know (3)
- Prefer Not To Answer (4)

***Display this question if C9 = 1***

**C9a** Were you diagnosed with… (check all that apply)

- Hepatitis A
- Hepatitis B
- Hepatitis C
- Don’t know
- Prefer Not To Answer

***Display this question if C6g AND C6h*** $\boldsymbol{\neq}$ ***8***

**C10** Have you ever been diagnosed with a sexually transmissible infection (STI)?

- Yes ______________ (1)
- No (2)
- Prefer Not To Answer (3)

**C11** Have you ever had an HIV antibody test (the test that tells whether a person is infected with HIV)?

- Yes (1)
- No (2)
- Don’t know (3)
- Prefer Not To Answer (4)

***Display this question if C11 = 1***

**C11a** When was the last time you had an HIV test?

- Within the last 3 months (1)
- 3-6 months ago (2)
- 6-12 months ago (3)
- More than 12 months ago (4)
- Don’t remember (5)
- Prefer Not To Answer (6)

***Peer Norms***

**These questions ask you about your perceptions of condom use.**

**C12** Do you think that people about the same age as you mostly use condoms if they have sex?

- I don’t think they have sex (1)
- None use condoms (2)
- A few do (3)
- About half do (4)
- Most of them do (5)
- All of them do (6)
- Prefer Not To Answer (7)

**C13** For those young people who use condoms when having sex, who do you think mostly suggests using a condom?

- Boys (1)
- Girls (2)
- Both (3)
- I don’t know (4)
- Prefer Not To Answer (5)

***Sexual Activity***

**Some people your age have had sex and other people have not. These questions ask you about your personal experiences of sex.**

**C14** Have you ever had a girlfriend or boyfriend?

- Yes (1)
- No (2)
- Don’t know/Not sure (3)
- Prefer Not To Answer (4)

**C15** Do you currently have a girlfriend or boyfriend?.

- Yes (1)
- No (2)
- Don’t know/Not sure (3)
- Prefer Not To Answer (4)

**C16** Have you ever had sex?.

- Yes (1)
- No (2)
- Prefer Not To Answer (3)

**C17** How old were you when you first had an experience of… Please select the age for each type of sexual experience.

**C17a-h options**

- Under 13 (1)
- 13 (2)
- 14 (3)
- 15 (4)
- 16 (5)
- 17 (6)
- 18 (7)
- Never (8)
- Prefer not to Answer (9)

C17a Deep kissing?

C17b Touching a partner’s genitals with your hands?

C17c Being touched on your genitals by a partner's hand?

C17d Touching your own genitals?

C17e Giving oral sex?

C17f Receiving oral sex?

C17g Anal sex?

C17h Vaginal sex?

***No Sexual Intercourse Display this section if C17g AND C17h = 8***

**C18a-i & C19a-I options**

- Not at all important (1)
- Slightly important (2)
- Moderately important (3)
- Very important (4)
- Extremely important (5)
- Prefer Not To Answer (6)

**C18** Here are some reasons that people may have for not having vaginal or anal intercourse. Please indicate how important these reasons are for you.

C18a I do not feel ready to have sexual intercourse

C18b My current partner (or last) is (was) not willing

C18c I am proud that I can say no and mean it

C18d It is against my religious beliefs

C18e It is against my cultural beliefs

C18f My fear of parental disapproval

C18g My fear of pregnancy

C18h It is important for me not to have sexual intercourse before I get married

C18i Fear of damaging my reputation

**C19** Here are some more reasons that people may have for not having vaginal or anal intercourse. Please indicate how important these reasons are for you.

C19a I have not met a person I wanted to have intercourse with

C19b I worry about contracting HIV/AIDS

C19c I worry about contracting STIs

C19d I am too shy or embarrassed to initiate sex with a partner

C19e I have not been in a relationship long enough

C19f It is important for me to be in love with the person with whom I first have sexual intercourse

C19g I do not feel physically attractive or desirable

C19h I have not had the opportunity to have vaginal/anal sex

C19i Other important reason(s) ________________

**C20** How likely are you to engage in vaginal or anal sex during the next year?

- Not at all likely (1)
- A little likely (2)
- Somewhat likely (3)
- Very likely (4)
- Extremely likely (5)
- Prefer Not To Answer (6)

**C21** How likely are you to engage in vaginal or anal sex before you get married?

- Not at all likely (1)
- A little likely (2)
- Somewhat likely (3)
- Very likely (4)
- Extremely likely (5)
- Prefer Not To Answer (6)

**C22** If you were in a close relationship with a partner who wanted to have vaginal or anal sex and the opportunity was available, would you have sex?

- Yes (1)
- No (2)
- Don’t know/Not sure (3)
- Prefer Not To Answer (4)

**C23** This question asks how you feel in relation to not having experienced vaginal or anal sex. Regarding not having experienced vaginal or anal sex, to what extent do you feel...

**C23a-k options**

- Not at all (1)
- A little (2)
- A fair amount (3)
- A lot (4)
- Extremely (5)
- Prefer Not To Answer (6)

C23a GOOD

C23b UPSET

C23c GUILTY

C23d HAPPY

C23e WORRIED

C23f REGRETFUL

C23g FANTASTIC

C23h ANXIOUS

C23i PROUD

C23j EMBARASSED

C23k OTHER FEELINGS ____________

**C24** This question asks about pressure you may have experienced to have sex.

**C24a-k options**

- No pressure (1)
- A little pressure (2)
- Some pressure (3)
- A fair amount of pressure (4)
- A lot of pressure (5)
- Not applicable (6)
- Prefer Not To Answer (7)

C24a How much pressure have you received from your girlfriend or boyfriend to have vaginal/anal sex with them?

C24b How much pressure have you received from peers or friends to have vaginal/anal sex?

C24c How much pressure have you received from parents/guardians/step-parents to remain a virgin?

C24d How much pressure have you received from peers or friends to remain a virgin?

***Had Sexual Intercourse Display this section if C17g AND C17h*** $\boldsymbol{\neq}$ ***8***

**C25** When you had vaginal or anal sex with people in the last year, how often were condoms used?

- Always used condoms (1)
- Often used condoms (2)
- Sometimes used condoms (3)
- Occasionally used condoms (4)
- Never used condoms (5)
- Prefer Not To Answer (6)

**Display this question if C17g**$\boldsymbol{\neq}$ ***8***

**C26** Did you use a condom the first time you had anal sex?

- Yes (1)
- No (2)
- Don’t remember (3)
- Prefer Not To Answer (4)

**Display this question if C17h**$\boldsymbol{\neq}$ ***8***

**C27** Did you use a condom the first time you had vaginal sex?

- Yes (1)
- No (2)
- Don’t remember (3)
- Prefer Not To Answer (4)

**C28** Think back to the last time you had sex. **BEFORE** you had vaginal and/or anal sex, did you talk to this person about…

**C28a-f options**

- Yes (1)
- No (2)
- Don’t Remember (3)
- Prefer Not To Answer (4)

C28a Avoiding pregnancy

C28b Avoiding HIV infection

C28c How to get sexual pleasure without intercourse

C28d Using a condom

C28e Having vaginal and/or anal sex

C28f Avoiding other sexually transmissible infections

**C29** Did you or the person with whom you had vaginal and/or anal sex have a condom with them/you the last time you had sexual intercourse?

- Yes (1)
- No (2)
- Don’t remember (3)
- Prefer Not To Answer (4)

**C30** Was a condom used the last time you had vaginal and/or anal sex?

- Yes (1)
- No (2)
- Don’t remember (3)
- Prefer Not To Answer (4)

***Display this question if C30 = 2***

**C30a** Why was a condom NOT used the last time you had sexual intercourse? Please select as many reasons as you think apply.

- I don’t like them (1)
- My partner doesn’t like them (2)
- I trust my partner (3)
- It just happened (4)
- We both have been tested for HIV/STIs (5)
- Too embarrassed (6)
- I know my partner’s sexual history (7)
- It is not my responsibility (8)
- Other _______________ (9)
- Prefer Not To Answer (10)

**Display this question if C17h**$\boldsymbol{\neq}$ ***8***

**C31** The last time you had vaginal sex which, if any, forms of contraception did you or the person you had sex with use to prevent pregnancy? Please select as many as you think apply.

- The pill (1)
- IUD (Intrauterine Device) (2)
- Diaphragm (3)
- Emergency Contraception (The morning after pill) (4)
- Withdrawal (5)
- Rhythm method (6)
- Condom (7)
- Injection (e.g., Depo-Provera) (8)
- Contraceptive Implant (e.g., Implanon) (9)
- None (10)
- Other _______________ (11)
- Prefer Not To Answer (12)

**C32** Have you ever had sex when you didn’t want to?

- Yes (1)
- No (2)
- Prefer Not To Answer (3)

***Display this question if C32 = 1***

**C32a** Below are some reasons why people had sex when they did not want to. Please check all that apply to you.

- I was too drunk at the time (1)
- I was too high at the time (2)
- My partner thought I should (3)
- My friends thought I should (4)
- I was frightened (5)
- Other __________________ (6)
- Prefer Not To Answer (7)

**Display this question if C17h**$\boldsymbol{\neq}$ ***8***

**C33** Have you ever had sex that resulted in a pregnancy?

- Yes (1)
- No (2)
- Don’t know (3)
- Prefer Not To Answer (4)

***Display this question if C33 = 1***

**C33a** Was the pregnancy planned?

- Yes (1)
- No (2)
- Prefer Not To Answer (3)

**C34** In the past year, who have you had sex with?

- Males only (1)
- Females only (2)
- Both males and females (3)
- Prefer Not To Answer (4)

**C35** Over the last year with how many people have you had anal and/or vaginal intercourse?

- 1 person (1)
- 2 people (2)
- 3 people (3)
- 4 or more people (4)
- Prefer Not To Answer (5)

**C36** Think back to the LAST TIME you had vaginal and/or anal sex. Was the last person you had vaginal and/or anal sex with…

- Someone you had just met for the first time? (1)
- Someone you had known for a while, but had not had sex with before? (2)
- Someone you had known for a while and had had sex with before, but not your girlfriend/boyfriend at the time? (3)
- Your girlfriend/boyfriend at the time? (4)
- Prefer Not To Answer (5)

**C37** Think back to the LAST TIME you had vaginal and/or anal sex. Was the last person you had vaginal and/or anal sex with…

- Male (1)
- Female (2)
- Other ________ (3)
- Prefer Not To Answer (4)

**C38** Think back to the LAST TIME you had vaginal and/or anal sex. How old was the last person you had vaginal and/or anal sex with?

- Under 16 years old (1)
- 16-17 years old (2)
- 18-19 years old (3)
- 20-24 years old (4)
- 25-29 years old (5)
- 30 years of age or older (6)
- Not sure (7)
- Prefer Not To Answer (8)

**C39** Think back to the LAST TIME you had vaginal and/or anal sex. When did you last have vaginal and/or anal sex with this person?

- In the last week (1)
- 1-3 weeks ago (2)
- 1-3 months ago (3)
- 4-6 months ago (4)
- 7-12 months ago (5)
- Over 12 months ago (6)
- Prefer Not To Answer (7)

**C40** Think back to the LAST TIME you had vaginal and/or anal sex. The last time you had vaginal and/or anal sex, where did this take place?

- My house (1)
- My girl/boy friend’s house (2)
- A friend’s house (3)
- Outside (e.g. park/beach) (4)
- In a car (5)
- Another place _________ (6)
- Prefer Not To Answer (7)

**C41** Were you drunk or high the last time you had sex?

- Yes (1)
- No (2)
- Prefer Not To Answer (3)

**C42** The last time you had sex did you want to have sex?

- Yes (1)
- No (2)
- Prefer Not To Answer (3)

**C43** The last time you had vaginal or anal sex, to what extent did you feel…

**C43a-k options**

- Not at all (1)
- A little (2)
- A fair amount (3)
- A lot (4)
- Extremely (5)
- Prefer Not To Answer (6)

C43a GOOD

C43b UPSET

C43c GUILTY

C43d HAPPY

C43e WORRIED

C43f REGRETFUL

C43g FANTASTIC

C43h ANXIOUS

C43i PROUD

C43j EMBARASSED

C43k OTHER FEELINGS ____________

***Technology Use Behaviours***

**The following questions ask you about your use of the Internet, technology and social media.**

**C44** In the last **2 MONTHS**, have you used any of the following social networking sites? Please select all that apply

- Facebook (1)
- Instagram (2)
- Snapchat (3)
- YouTube (4)
- Twitter (5)
- LinkedIn (6)
- GooglePlus (7)
- Tumblr (8)
- Pinterest (9)
- Reddit (10)
- Vine (11)
- MySpace (12)
- Flickr (13)
- Dating App such as Tindr (Please specify which one you used the most in the last 2 months) _____________ (14)
- I haven’t used any of these (15)
- Other – please specify _______ (16)
- Prefer Not To Answer (17)

***Display this question if C44*** $\boldsymbol{\neq}$ ***17 AND at least one option selected***

***(only mirror statements presented)***

**C44a** In the last **2 MONTHS**, how often have you used…

**C44a1-k options**

- More than 5 times a day (1)
- Every day / most days (2)
- A few times a week (3)
- About once a week (4)
- Less than once a week (5)
- Prefer Not To Answer (6)

C44a1 Facebook

C44a2 Instagram

C44a3 Snapchat

C44a4 YouTube

C44a5 Twitter

C44a6 LinkedIn

C44a7 GooglePlus

C44a8 Tumblr

C44a9 Pinterest

C44a10 Reddit

C44a11 Vine

C44a12 MySpace

C44a13 Flickr

C44a14 Dating App (fill-in-the-blank from C44 presented as option)

C44a16 Other (fill-in-the-blank from C44 presented as option)

**C45** In the past **2 MONTHS**, have any of the following happened?

**C45a-f options**

- Yes (1)
- No (2)
- Don’t know / not sure (3)
- Prefer Not To Answer (4)

C45a You sent a sexually explicit written text message

C45b You received a sexually explicit written text message

C45c You sent a sexually explicit nude or nearly nude photo or video of yourself

C45d You sent a sexually explicit nude or nearly nude photo or video of someone else

C45e You received a sexually explicit nude or nearly nude photo or video of someone else

C45f You used a social media site for sexual reasons

***Display these questions (C45a & C45b) if any of C45a-f = 1***

***(only mirror statements presented)***

**C45a** In the past **2 MONTHS**, how often have you…

**C45a1-6 options**

- Once a day or more (1)
- A few times a week (2)
- About once a week (3)
- A few times a month (4)
- Only once in the past 2 months (5)
- Prefer Not To Answer (6)

C45a1 Sent a sexually explicit written text message?

C45a2 Received a sexually explicit written text message?

C45a3 Sent a sexually explicit nude photo or video of yourself?

C45a4 Sent a sexually explicit nude photo or video of someone else?

C45a5 Received a sexually explicit nude photo or video of someone else?

C45a6 Used a social media site for sexual reasons?

**C45b** If **YES**, In the past **2 MONTHS** (please check all that apply)…

**C45a1-6 options**

- Boyfriend (1)
- A good friend (2)
- Someone who is not a close friend (3)
- Someone around my age who I just met (4)
- Someone not around my age who I just met (5)
- Stranger (6)
- Prefer Not To Answer (7)

C45b1 To whom have you sent sexually explicit written text messages?

C45b2 From whom have you received sexually explicit written text messages?

C45b3 To whom did you send sexually explicit nude or nearly nude photos or videos of yourself?

C45b4 To whom did you send sexually explicit nude or nearly nude photos or videos of someone else?

C45b5 From whom did you receive sexually explicit nude or nearly nude photos or videos of someone else?

C45b6 With whom have you used a social media site for sexual reasons?

**C46** In the past **2 MONTHS**, which of these things have happened to you? Please tick all that apply.

- I was sent threatening emails (1)
- I was sent nasty messages on the Internet, e.g. through Facebook Chat, Skype, Tumblr (2)
- I was sent nasty text messages (3)
- I received prank calls on my mobile phone (4)
- Someone used my username or profile, pretending to be me to hurt someone else (5)
- Someone sent my private emails, messages, pictures or videos to others (6)
- Mean or nasty comments or pictures were sent or posted about me to websites, e.g. Facebook, Twitter or Tumblr (7)
- Mean or nasty messages or pictures were sent about me to other students’ mobile phones (8)
- I was deliberately ignored or left out of things over the Internet (9)
- Other _______________________________________________ (10)
- None of these happened to me (11)
- Prefer not to answer (12)

***Display this question if C46*** $\boldsymbol{\neq}$ ***11 or 12 AND at least one option selected***

***(only mirror statements presented)***

**C46a** If **YES**, in the past 2 MONTHS, how often…

**C46a1-11 options**

- Once a day or more (1)
- A few times a week (2)
- About once a week (3)
- A few times a month (4)
- Only once in the past 2 months (5)
- Prefer Not To Answer (6)

C46a1 Were you sent threatening emails?

C46a2 Were you sent nasty messages on the Internet (e.g. through Facebook Chat, Skype, Tumblr)?

C46a3 Were you sent nasty text messages?

C46a4 Did you receive prank calls on your mobile phone?

C46a5 Did someone use your username or profile, pretending to be you to hurt someone else?

C46a6 Did someone send your private emails, messages, pictures or videos to others?

C46a7 Were mean or nasty comments or pictures sent or posted about you to websites, e.g. Facebook, Twitter or Tumblr?

C46a8 Were mean or nasty messages or pictures sent about you to other students’ mobile phones?

C46a9 Were you deliberately ignored or left out of things over the Internet?

C46a10 Did you experience [Other]? (fill-in-the-blank from C46 presented as option)

**D: EDUCATION**

***Informal Education***

**This section asks you about talking to others and the way/s in which you have been taught about sex and sexual health.**

**D1** How confident are you that you could talk about **HIV and other Sexually Transmissible Infections** with…

**D2** How confident are you that you could talk about **decisions concerning contraception** with…

**D3** How confident are you that you could talk about **sex** with…

**D1-3 were asked for each of the following persons:**

1. Doctor/GP
2. School counsellor
3. School nurse
4. Teacher
5. Youth worker
6. Mother/female guardian/step-parent
7. Father/male guardian/step-parent
8. Female friend
9. Male friend
10. Older brother/sister
11. Other _______________

**D1-3 response options**

- Not at all confident (1)
- Not very confident (2)
- Somewhat confident (3)
- Confident (4)
- Very confident (5)
- Prefer Not To Answer (6)

**D4, D5, & D5a were asked about each of the following sources of information:**

1. Doctor/GP
2. School counsellor
3. School nurse
4. Teacher
5. Youth worker
6. Mother/female guardian/step-parent
7. Father/male guardian/step-parent
8. Female friend
9. Male friend
10. Older brother/sister
11. Internet website
12. School program
13. Community health service
14. Other _______________

**D4** For each of the following, please indicate how much you trust them to provide you with accurate sexual health information.

**D4a-n options**

- Do not trust (1)
- Trust a little (2)
- Somewhat trust (3)
- Trust (4)
- Trust a lot (5)
- Not sure (6)
- Prefer Not To Answer (7)

**D5** Please select all of the sources of information below that you have **ever** used for advice about sexual health.

**D5a-n options**

- Used (1)
- Not used (2)
- Prefer Not To Answer (3)

***Display this question if D5 = 1***

***(only mirror statements presented)***

**D5a** In the **past year**, how often have you used the following for advice about **sexual health**?

D5a a-n options

- Not used in the past year (1)
- Once or twice (2)
- A few times (3)
- Several times (4)
- About once a month (5)
- More than once a month (6)
- Almost weekly (7)
- Prefer Not To Answer (8)

***Formal Education***

**D6** Have you ever had sexuality/relationship education at school?

- Yes (1)
- No (2)
- Don’t know/Not sure (3)
- Prefer Not To Answer (4)

***Display these question (D6a-d) if D6 = 1***

**D6a** At what levels of schooling did you have sexuality/relationship education?

Please select as many as you think apply.

- Prep/kindergarten (1)
- Years 1-4 (2)
- Years 5-6 (3)
- Years 7-8 (4)
- Years 9-10 (5)
- Years 11-12 (6)
- Prefer Not To Answer (7)

**D6b** Thinking back to the **last time** you had sexuality/relationship education, what subject was it part of?

- It was its own subject (1)
- Health and Physical Education (2)
- Science/Biology (3)
- Religious Instruction/Education (4)
- Other – please specify________ (5)
- Prefer Not To Answer (6)

**D6c** Thinking back to the **last time** you had sexuality / relationship education, who was it primarily taught by?

- A teacher (1)
- A school nurse (2)
- A chaplain (3)
- School counsellor (4)
- Someone from outside the school ____________ (5)
- Someone else ___________ (6)
- Prefer Not To Answer (7)

**D6d** How relevant did you/do you find sexuality/relationship classes?

- Not relevant at all (1)
- A little relevant (2)
- Somewhat relevant (3)
- Very relevant (4)
- Extremely relevant (5)
- Prefer Not To Answer (6)

**D7** Is there is anything you would like to tell us about sexuality education at your school (e.g., how useful it has been for you)?

Please write your comments here or type "No Comment".

………………………………………………………………………………………………………………………………………………………………………………………………………
